# Exome sequencing reveals a high prevalence of *BRCA1* and *BRCA2* founder variants in a diverse population-based biobank

**DOI:** 10.1186/s13073-019-0691-1

**Published:** 2019-12-31

**Authors:** Noura S. Abul-Husn, Emily R. Soper, Jacqueline A. Odgis, Sinead Cullina, Dean Bobo, Arden Moscati, Jessica E. Rodriguez, CBIPM Genomics Team, Regeneron Genetics Center, Ruth J. F. Loos, Judy H. Cho, Gillian M. Belbin, Sabrina A. Suckiel, Eimear E. Kenny

**Affiliations:** 1 The Center for Genomic Health, Icahn School of Medicine at Mount Sinai, New York, NY USA; 2 The Charles Bronfman Institute for Personalized Medicine, Icahn School of Medicine at Mount Sinai, New York, NY USA; 3 Department of Medicine, Icahn School of Medicine at Mount Sinai, New York, NY USA; 4 Department of Genetics and Genomic Sciences, Icahn School of Medicine at Mount Sinai, New York, NY USA; 5 Regeneron Genetics Center, Tarrytown, New York, NY USA

## Abstract

**Background:**

Pathogenic variants in *BRCA1* and *BRCA2* (*BRCA1/2*) lead to increased risk of breast, ovarian, and other cancers, but most variant-positive individuals in the general population are unaware of their risk, and little is known about prevalence in non-European populations. We investigated *BRCA1/2* prevalence and impact in the electronic health record (EHR)-linked Bio*Me* Biobank in New York City.

**Methods:**

Exome sequence data from 30,223 adult Bio*Me* participants were evaluated for pathogenic variants in *BRCA1/2*. Prevalence estimates were made in population groups defined by genetic ancestry and self-report. EHR data were used to evaluate clinical characteristics of variant-positive individuals.

**Results:**

There were 218 (0.7%) individuals harboring expected pathogenic variants, resulting in an overall prevalence of 1 in 139. The highest prevalence was in individuals with Ashkenazi Jewish (AJ; 1 in 49), Filipino and other Southeast Asian (1 in 81), and non-AJ European (1 in 103) ancestry. Among 218 variant-positive individuals, 112 (51.4%) harbored known founder variants: 80 had AJ founder variants (*BRCA1* c.5266dupC and c.68_69delAG, and *BRCA2* c.5946delT), 8 had a Puerto Rican founder variant (*BRCA2* c.3922G>T), and 24 had one of 19 other founder variants. Non-European populations were more likely to harbor *BRCA1/2* variants that were not classified in ClinVar or that had uncertain or conflicting evidence for pathogenicity (uncertain/conflicting). Within mixed ancestry populations, such as Hispanic/Latinos with genetic ancestry from Africa, Europe, and the Americas, there was a strong correlation between the proportion of African genetic ancestry and the likelihood of harboring an uncertain/conflicting variant. Approximately 28% of variant-positive individuals had a personal history, and 45% had a personal or family history of *BRCA1/2-*associated cancers. Approximately 27% of variant-positive individuals had prior clinical genetic testing for *BRCA1/2*. However, individuals with AJ founder variants were twice as likely to have had a clinical test (39%) than those with other pathogenic variants (20%).

**Conclusions:**

These findings deepen our knowledge about *BRCA1/2* variants and associated cancer risk in diverse populations, indicate a gap in knowledge about potential cancer-related variants in non-European populations, and suggest that genomic screening in diverse patient populations may be an effective tool to identify at-risk individuals.

## Background

The recognition of strong familial clustering of breast and ovarian cancer [1], followed by the discovery of the *BRCA1* and *BRCA2* (*BRCA1/2*) genes in 1994 [2] and 1995 [3], respectively, has led to the study and characterization of *BRCA1/2*-related hereditary breast and ovarian cancer syndrome (HBOC). Inherited pathogenic variants in either of these genes cause a significantly elevated risk for cancer of the female breast as well as high-grade serous ovarian, tubal, and peritoneal carcinoma. The risk for other cancers, including prostate, male breast, pancreas, melanoma and possibly others, is also increased [4]. Pathogenic variants in these genes are highly penetrant and inherited in an autosomal dominant pattern.

The prevalence of pathogenic *BRCA1/2* variants has been previously estimated, with historical data suggesting a prevalence of approximately 1 in 400 individuals in the general population [5, 6]. A higher prevalence has been observed in certain populations; for example, approximately 1 in 42 individuals of Ashkenazi Jewish (AJ) descent harbor one of three common founder variants [7, 8]. Founder variants in other populations have also been described, including Icelandic, French Canadian, and Puerto Rican populations [9]. Recent unselected population-based genomic screening efforts have demonstrated a higher than expected prevalence of *BRCA1/2* pathogenic variants in predominantly European-ancestry individuals, approximately 1 in 190, with only half of these individuals meeting current guidelines for genetic testing [10–12] and only 18% having prior knowledge of their *BRCA1/2* status through clinical genetic testing [13].

Understanding of the prevalence and contribution to cancer risk of *BRCA1/2* variants in non-European populations has been limited by racial and ethnic disparities in genetic research [14]. In addition to reduced uptake of genetic testing in diverse populations [15–18], there is a higher rate of detection of variants of uncertain significance in non-European populations [19–21]. Here, we evaluated the range of *BRCA1/2* variants in a diverse patient population from the Bio*Me* Biobank in New York City and explored clinical characteristics of individuals harboring expected pathogenic variants in *BRCA1/2*.

## Methods

### Setting and study population

The Bio*Me* Biobank is an electronic health record (EHR)-linked biobank of over 50,000 participants from the Mount Sinai Health System (MSHS) in New York, NY. Participant recruitment into Bio*Me* has been ongoing since 2007 and occurs predominantly through ambulatory care practices across the MSHS. The Bio*Me* participants in this analysis were recruited between 2007 and 2015, with approximately half coming from general medicine and primary care clinics and the rest from different specialty or multi-specialty sites at MSHS. Bio*Me* participants consent to provide DNA and plasma samples linked to their de-identified EHRs. Participants provide additional information on self-reported ancestry, personal and family medical history through questionnaires administered upon enrollment. This study was approved by the Icahn School of Medicine at Mount Sinai’s Institutional Review Board. The study population consisted of 30,223 consented Bio*Me* participants aged 18 years or older (upon enrollment) and with exome sequence data available through a collaboration with the Regeneron Genetics Center.

### Generation and QC of genomic data

Sample preparation and exome sequencing were performed at the Regeneron Genetics Center as previously described [22] yielding *N* = 31,250 samples and *n* = 8,761,478 sites. Genotype array data using the Illumina Global Screening Array was also generated for each individual [23]. Post-hoc filtering of the sequence data included filtering of *N* = 229 low-quality samples, including low-coverage, contaminated, and genotype-exome discordant samples; *N* = 208 gender discordant and duplicate samples were also removed. This resulted in *N* = 30,813 samples for downstream analysis, and *N* = 30,223 samples from participants aged 18 years and older. Mean depth of coverage for the remaining samples was 36.4x, and a minimum depth of 27.0x, and sequence coverage was sufficient to provide at least 20x haploid read depth at > 85% of targeted bases in 96% of samples. Sites with missingness greater than 0.02 (*n* = 267,955 sites) were removed, as were sites showing allele imbalance (*n* = 320,877; allelic balance < 0.3 or > 0.8). Samples were stratified by self-reported ancestry, and sites with Hardy Weinberg equilibrium *p* < 1 × 10^− 6^ (*n* = 12,762) were removed from analysis. Variants at multi-allelic sites in *BRCA1* and *BRCA2* (*n* = 124) underwent the same quality control workflow as those from bi-allelic sites, with the exception that allelic balance was calculated only among heterozygous carriers of multi-allelic variants. Multi-allelic sites for which the mean allelic balance among heterozygous carriers was < 0.3 or > 0.8 were excluded from downstream analysis. This resulted in the exclusion of *n* = 1 site, leaving a total of *n* = 123 for further analysis. Manual inspection of pileups was performed for carriers (*N* = 22) of the *n* = 13 multi-allelic sites annotated as pathogenic in ClinVar. Of these, *N* = 6 out of 7 carriers of the 13:32339421:C:CA variant were determined to be false positives and excluded from downstream analyses.

### Self-reported and genetic ancestry

Self-reported ancestry categories were derived from a multiple-choice survey administered to participants upon enrollment into the Bio*Me* Biobank [23]. Participants could select one or more of the following categories: African American/African, American Indian/Native American, Caucasian/White, East/Southeast Asian, Hispanic/Latino, Jewish, Mediterranean, South Asian/Indian, or Other. Individuals who selected “Jewish,” “Caucasian/White,” or both were designated as “European.” Individuals who selected “Mediterranean,” “Other,” or both were designated as “Other.” Individuals who selected multiple categories including “Hispanic/Latino” were designated as “Hispanic/Latino.” Individuals from the “Native American,” “Other,” or “Multiple Selected” categories were excluded from downstream analysis of prevalence in self-reported groups.

Genetic ancestry in the form of identity-by-descent community designation was performed on a subset of participants excluding second-degree relatives and above, yielding 17 distinct communities representing patterns of cultural endogamy and recent diaspora to New York City. Eight of these communities with > 400 unrelated participants were used for downstream analysis of prevalence. These communities included individuals with African American and African ancestry (*N* = 6874), non-AJ European ancestry (*N* = 5474), AJ ancestry (*N* = 3887), Filipino and other Southeast Asian ancestry (*N* = 556), as well as ancestry from Puerto Rico (PR; *N* = 5105), the Dominican Republic (DR; *N* = 1876), Ecuador (*N* = 418), and other Central and South American communities (*N* = 1116). Full details of the global ancestry inference, genetic community detection, and genotype quality control are described in Belbin et al. [23]. Finally, we determined the proportion African genetic ancestry in mixed ancestry Hispanic/Latino populations using the ADMIXTURE [24] software. We assumed five ancestral populations (*k* = 5) with 5-fold cross validation across *n* = 256,052 SNPs in *N* = 27,984 unrelated participants that were also genotyped on the Global Screening Array (GSA), in addition to *N* = 4149 reference samples representing 5 continental regions [23]. Unrelated, self-reported Hispanic/Latino participants with both exome sequence and GSA genotype data (*N* = 8457) were extracted, and binned into four groups of proportion African genetic ancestry; 0-20% (*N*= 3748), >20-40% (*N* = 2779), >40-60% (*N* = 1242), and >60% (*N* = 688). We estimated relatedness using the software KING [25], and for all prevalence estimates in self-reported and genetic ancestry groups, we excluded second-degree relatives and above.

### *BRCA1/2* variant annotation

Sequence variants were annotated with the Variant Effect Predictor (VEP; Genbank gene definitions; *BRCA1* NM_007294.3, *BRCA2* NM_000059.3). In order to reduce the set of false positive predicted loss-of-function (pLOF) calls, we also ran the Loss-Of-Function Transcript Effect Estimator (LOFTEE) and defined the consensus calls from both methods as the set of pLOF variants for the study. Sequenced variants were cross-referenced with the ClinVar database (accessed July 2018) [26] and annotated according to their ClinVar assertions when available as pathogenic, likely pathogenic, uncertain significance, benign, likely benign, or with conflicting interpretations of pathogenicity. All variants with conflicting interpretations were manually reviewed in ClinVar (accessed November 2018) by a genetic counselor (J.A.O. or E.R.S.). In addition, we included the following categories of pLOF variants not classified in ClinVar: single nucleotide variants (SNVs) leading to a premature stop codon, loss of a start codon, or loss of a stop codon; SNVs or insertion/deletion sequence variants (indels) disrupting canonical splice acceptor or donor dinucleotides; and open reading frame shifting indels leading to the formation of a premature stop codon. The union of ClinVar pathogenic/likely pathogenic and pLOF variants was termed “expected pathogenic,” and this set of variants was used to identify individuals in Bio*Me* for subsequent analyses of HBOC-related clinical characteristics.

### *BRCA1/2* founder variants

All expected pathogenic variants detected in *BRCA1/2* were reviewed for evidence of a founder effect. This was carried out by manual review of each expected pathogenic variant by a genetic counselor (E.R.S.) in the Human Gene Mutation Database [27], ClinVar, and PubMed utilizing the currently designated HGVS nomenclature for each variant [28], as well as previous designations as noted in ClinVar. Variants were considered to be founder variants if they were described as such in the primary literature, based on confirmatory haplotype analysis or population frequency.

### Clinical characteristics in variant-positive individuals

Individuals harboring expected pathogenic variants in *BRCA1/2* in Bio*Me*, termed “variant positive,” were evaluated for any evidence of personal or family histories of HBOC-related cancers, through extraction of International Classification of Diseases (ICD)-9 and ICD-10 codes from participant EHRs (Additional file 1: Table S1). These data were supplemented by participant questionnaire data for personal and family histories of HBOC-related cancers, which were available for 61 variant-positive individuals. Medical record review of variant-positive individuals was carried out independently by two individuals, including genetic counselors (J.A.O., E.R.S., or S.A.S.) and a clinical research coordinator (J.E.R.) to determine whether participants had evidence of previous clinical genetic testing for *BRCA1/2*. Data were summarized using medians and interquartile ranges (IQR) for continuous variables and frequencies and percentages for categorical variables. Pearson’s chi-squared test with Yates correction was used to test for statistical independence of different categorical outcomes measured in the study.

### HBOC-related cancer case-control and phenome-wide association studies

Cases were defined as participants having any of the ICD-9 or ICD-10 codes for personal history of HBOC-related cancers (Additional file 1: Table S1). Controls were defined as individuals without any of these ICD-9 or ICD-10 codes. We tested for association with variant-positive compared with variant-negative participants (defined as not having any variants that were pathogenic, uncertain/conflicting, or unclassified in ClinVar (novel)). Genotypes were coded using a binary model (0 for variant negative and 1 for variant positive). We repeated the analysis to compare participants with uncertain/conflicting variants with variant-negative participants. We excluded individuals determined to be second-degree relatives and above from the analysis. Odds ratios were estimated by logistic regression and adjusted for age, sex, and the first 5 principal components of ancestry.

We also performed a phenome-wide association study (PheWAS) of variant-positive vs. variant-negative participants using ICD-9- and ICD-10-based diagnosis codes that were collapsed to hierarchical clinical disease groups (termed phecodes) [29, 30]. We performed logistic regression systematically using *BRCA1/2* expected pathogenic carrier status as the primary predictor variable and the presence of a given phecode as the outcome variable, excluding second-degree relatives and above and adjusting for age, sex, and the first 5 principal components. To minimize spurious associations due to limited numbers of case observations, we restricted analyses to phecodes present in at least 5 variant-positive participants, resulting in a total of *p* = 260 tests. Statistical significance was determined using Bonferroni correction (Bonferroni-adjusted significance threshold *p* < 1.9 × 10^− 4^). Logistic regression analyses were performed using PLINK (v1.90b3.35) software.

## Results

**Fig. 1:**
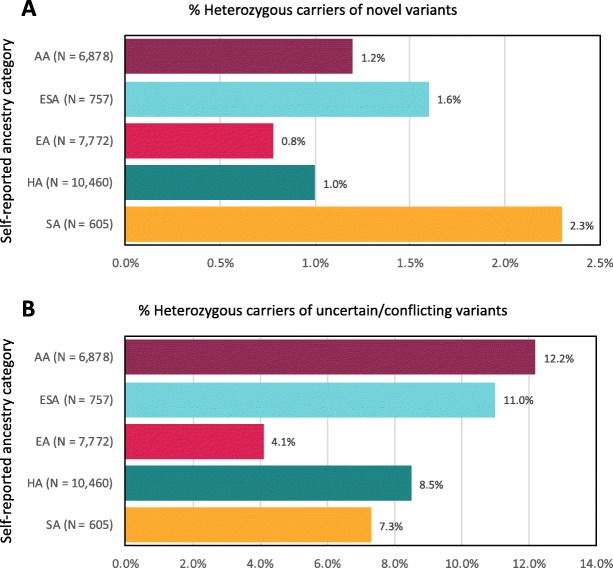
Among 1601 *BRCA1/2* variants identified in the Bio*Me* Biobank, there were 266 variants not classified in ClinVar (novel) and 635 variants of uncertain significance or with conflicting interpretations of pathogenicity in ClinVar (uncertain/conflicting). The proportion of individuals harboring novel (**a**) or uncertain/conflicting (**b**) variants varied across self-reported ancestry categories and was lowest among individuals of European descent (0.8% and 4.1%, respectively). The proportion of individuals harboring novel variants was highest in individuals of South Asian descent (2.3%), and the proportion harboring uncertain/conflicting variants was highest in individuals of African American/African descent (12.2%). AA, African American/African descent; ESA, East/Southeast Asian descent; EA, European descent; HA, Hispanic/Latino descent; SA, South Asian descent

We evaluated *BRCA1/2* variants among 30,223 adult participants of the Bio*Me* Biobank with available exome sequence data and genotype array data. Participants were 59.3% female and had a median age of 59 years (Table 1). The majority of participants (74.3%) were of non-European descent, based on self-report. A total of 1601 variants were analyzed, including 1478 (92.3%) occurring at bi-allelic sites and 123 (7.7%) at multi-allelic sites. The majority of variants were missense (63.5%), and 1335 (83.4%) variants were available in ClinVar (Additional file 1: Table S2). The proportion of individuals harboring *BRCA1/2* variants that were not classified in ClinVar (novel) was lowest in individuals of self-reported European descent (0.8%) and highest in individuals of South Asian descent (2.3%; Fig. 1a). The proportion of individuals harboring *BRCA1/2* variants of uncertain significance or with conflicting interpretations of pathogenicity (uncertain/conflicting) in ClinVar was lowest in individuals of self-reported European descent (4.1%) and highest in those of self-reported African American/African descent (12.2%; Fig. 1b). We saw a similar trend when investigating genetic ancestry within populations with recent mixed ancestry, for example, Hispanic/Latino populations, who can trace their recent ancestry to Europe, Africa, and the Americas (Additional file 1: Figure S1). Although the mean uncertain/conflicting variant rate in all self-reported Hispanic/Latino participants was 8.5% (95% CI 7.9-9.1%; Fig. 1b), this rate was almost twofold higher in those with > 60% African genetic ancestry (11.3% (95% CI 9.2–13.9%)) compared with those with < 20% African genetic ancestry (6.9% (95% CI 6.1–7.7%); chi-squared *p* = 7.8 × 10^− 5^; Additional file 1: Figure S1).

**Table 1:** Demographics of exome-sequenced adult Bio*Me* Biobank participants and of individuals harboring expected pathogenic variants in *BRCA1/2*

	Sequenced Bio*Me* participants (*N* = 30,223)	*BRCA1/2* variant negative (*N* = 27,060)*	*BRCA1/2* variant positive (*N* = 218)
Age, median (IQR)	59 (45–70)	59 (46–70)	58 (43–70)
Female, *N* (%)	17,914 (59.3)	15,986 (59.1)	137 (62.8)
Self-reported ancestry, *N* (%)			
African American/African	6878 (22.8)	5877 (28.3)	33 (15.1)
East/Southeast Asian	757 (2.5)	659 (3.2)	6 (2.8)
European	7772 (25.7)	7265 (35.0)	121 (55.8)
Hispanic/Latino	10,460 (34.6)	9360 (45.1)	34 (15.6)
Native American	52 (0.2)	47 (0.2)	0 (0)
South Asian	605 (2.0)	543 (2.6)	0 (0)
Other	2343 (7.8)	2111 (10.2)	13 (6.0)
Multiple selected	1125 (3.7)	1006 (4.9)	10 (4.6)
Not available	231 (0.8)	192 (0.9)	1 (0.5)

*Variant-negative participants are defined as not having any variants that were pathogenic, uncertain/conflicting, or unclassified in ClinVar

Exome sequence data of the *BRCA1/2* genes was then used to identify expected pathogenic variants. There were 102 variants with a pathogenic or likely pathogenic assertion in ClinVar, all of which had a 2- or 3-star review status (Additional file 1: Table S3). There were 10 additional pLOF variants (frameshift or stop gained) that were not classified in ClinVar, including 2 in *BRCA1* and 8 in *BRCA2*. The 10 pLOF variants were each observed as singletons in Bio*Me*, and only one of them (*BRCA2* c.1039C>T) was found in the gnomAD database [31] with an allele frequency of 0.000004, suggesting that these are rare in the general population. The union of 102 ClinVar pathogenic and 10 additional rare pLOF variants was the set of expected pathogenic *BRCA1/2* variants (*n* = 112) used to define variant-positive individuals in Bio*Me*.

Overall, 218 (0.7%) individuals in Bio*Me* harbored expected pathogenic variants in *BRCA1/2*: 86 (39.4%) of these individuals had an expected pathogenic variant in *BRCA1*, 131 (60.1%) had a variant in *BRCA2*, and 1 (0.5%) individual had a variant in both *BRCA1* (c.68_69delAG) and *BRCA2* (c.5946delT). Variant-positive individuals were 62.8% female and had a median age of 58 years (Table 1). The prevalence of Bio*Me* participants harboring expected pathogenic variants in *BRCA1/2* was 1:139 (Table 2). In a subset of individuals excluding second-degree relatives and above (*N* = 27,816), overall prevalence was unchanged at 1:134. In the unrelated subset, prevalence was highest in individuals of self-reported European descent (1:66) and lowest in those of Hispanic/Latino descent (1:283). We previously used genotype array data to identify fine-scale population groups in Bio*Me* using genetic ancestry [23], revealing eight communities with greater than 400 individuals represented (Table 2). Across these, prevalence was highest in individuals with AJ ancestry (1:49), among whom the majority (72 out of 80 individuals, or 90.0%) harbored one of the three AJ founder variants (c.5266dupC and c.68_69delAG in *BRCA1*, and c.5946delT in *BRCA2*), and 8 individuals (10.0%) harbored a different variant in *BRCA1/2* (Additional file 1: Table S3). Prevalence was lower in non-AJ Europeans (1:103) and lowest in those with ancestry from PR (1:340) and DR (1:469; Table 2).

**Table 2:** Prevalence of expected pathogenic *BRCA1/2* variants in the Bio*Me* Biobank. We assessed the prevalence of *BRCA1/2* variants in all sequenced participants, in an unrelated subset of participants, across self-reported ancestry groups, and across genetic ancestry groups for which there were greater than 400 individuals

Population characteristics	*N*	*BRCA1/2* variant positive, *N* (%)	Estimated prevalence
All sequenced participants	30,223	218 (0.7)	1:139
Unrelated subset—including only one individual in every first- and second-degree relationship	27,816	208 (0.7)	1:134
Self-reported ancestry (unrelated subset)			
African American/African	6236	31 (0.5)	1:201
East/Southeast Asian	739	6 (0.8)	1:123
European	7600	116 (1.5)	1:66
Hispanic/Latino	9050	32 (0.4)	1:283
Native American	47	0 (0)	–
South Asian	585	0 (0)	–
Other	2271	13 (0.6)	–
Multiple selected	1078	9 (0.8)	–
Not available	211	1 (0.5)	–
Genetic ancestry (unrelated subset)			
African American and African	6874	31 (0.5)	1:222
Ashkenazi Jewish	3889	80 (2.1)	1:49
Non-Ashkenazi Jewish European	5474	53 (1.0)	1:103
Filipino and other Southeast Asian	566	7 (1.2)	1:81
Dominican	1876	4 (0.2)	1:469
Ecuadorian	418	2 (0.5)	1:209
Puerto Rican	5105	15 (0.3)	1:340
Other Central and South American	1116	8 (0.7)	1:140

We identified 23 unique founder variants that have previously been reported in multiple founder populations, including 13 variants in *BRCA1* and 10 in *BRCA2* (Table 3). A total of 112 of 218 variant-positive individuals (51.4%) were identified as harboring at least one founder variant (61 individuals with a variant in *BRCA1*, 50 with *BRCA2*, and 1 with both *BRCA1* and *BRCA2*). The majority of identified founder variants were accounted for by the three AJ founder variants, with 80 individuals in Bio*Me* harboring at least one of these variants, 72 of whom had AJ genetic ancestry. There were 32 participants harboring non-AJ founder variants in *BRCA1/2*, the most common being *BRCA2* c.3922G>T, a well-documented founder variant in PR [47]. Among 15 *BRCA1/2* variant-positive individuals with genetic ancestry from PR, 7 (46.7%) harbored the *BRCA2* c.3922G>T variant, and 3 others (20.0%) harbored Chilean or Spanish founder variants (Table 3).

**Table 3:** Founder variants identified among 112 *BRCA1/2* expected pathogenic variants in the Bio*Me* Biobank

Gene	cDNA position^#^	Bio*Me* self-reported ancestry (# Hets)	Bio*Me* genetic ancestry (# Hets)	Previously described founder population (reference)
*BRCA1*	c.5335delC	ESA (1)	Filipino and other Southeast Asian (1)	Filipino [32]
*BRCA1*	c.5266dupC	EA (6)	AJ (5), non-AJ European (1)	AJ [8, 33]
*BRCA1*	c.5123C>A	EA (1)	Non-AJ European (1)	Columbian, Spanish [34]
*BRCA1*	c.4327C>T	O (1)	Non-AJ European (1)	French Canadian [35], Mexican, Columbian, Peruvian [36]
*BRCA1*	c.3817C>T	HA (1)	Puerto Rican (1)	Chilean [37]
*BRCA1*	c.3756_3759delGTCT	EA (2)	Non-AJ European (1)	French Canadian* [38]
*BRCA1*	c.3331_3334delCAAG	AA (1), HA (1)	African American and African (1), other Central and South American (1)	Colombian [34], Chilean [37]
*BRCA1*	c.2475delC	EA (1)	AJ (1)	Scandinavian* [39]
*BRCA1*	c.303 T>G	AA (1)	African American and African (1)	African [40]
*BRCA1*	c.211A>G	HA (2), ESA (1)	Puerto Rican (2), Filipino and other Southeast Asian (1)	Spanish [41]
*BRCA1*	c.181 T>G	EA (1)	Non-AJ European (1)	Polish* [42]
*BRCA1*	c.116G>A	M (1)		Italian [43]
*BRCA1*	c.68_69delAG	EA (36), M (4), O (1)	AJ (38)	AJ [8, 44]
*BRCA2*	c.2808_2811delACAA	HA (1)	Other Central and South American (1)	Western European [45], Columbian [46]
*BRCA2*	c.3922G>T	HA (8)	Puerto Rican (7)	Puerto Rican [47]
*BRCA2*	c.4631delA	O (1)	Filipino and other Southeast Asian (1)	Filipino [32]
*BRCA2*	c.5351dupA	M (1)	Non-AJ European (1)	Dutch [45]
*BRCA2*	c.5576_5579delTTAA	ESA (1)	Filipino and other Southeast Asian (1)	Japanese [48]
*BRCA2*	c.5857G>T	AA (1)	African American and African (1)	French Canadian [49]
*BRCA2*	c.5946delT	EA (30), M (1), NA (1), O (2)	AJ (30), non-AJ European (1)	AJ [8, 45]
*BRCA2*	c.6644_6647delACTC	HA (1)	African American and African (1)	French* [50]
*BRCA2*	c.7480C>T	EA (1), HA (1)	Non-AJ European (1), Dominican (1)	Korean [51], Finnish [52]
*BRCA2*	c.7913_7917delTTCCT	EA (1)	AJ (1)	Czech* [53]

Abbreviations: *Hets* heterozygous carriers, *AA* African American/African, *AJ* Ashkenazi Jewish, *EA* European, *ESA* East/Southeast Asian, *HA* Hispanic/Latino, *M* multiple selected ancestries, *NA* not available, *O* other self-reported ancestry

^#^cDNA position provided for *BRCA1* ENST00000357654 (NM_007294.3) and *BRCA2* ENST00000380152 (NM_000059.3)

*Variant described in literature as a founder variant, but no haplotype evidence available

We evaluated the clinical characteristics of *BRCA1/2* variant-positive individuals using EHR-extracted diagnosis codes (Additional file 1: Table S1), as well as additional personal and family medical history questionnaire data available for 61 of these individuals. Overall, 61 of 218 (28.0%) *BRCA1/2* variant-positive individuals had a documented personal history and 98 (45.0%) had either a personal or family history of HBOC-related cancer (breast, ovarian, pancreatic, prostate, or melanoma; Table 4). Variant-positive females were 2.8 times more likely than males to have a personal or family history of HBOC-related cancers (chi-squared *p* = 9.9 × 10^− 8^). Among variant-positive females (*N* = 137), 53 (38.7%) had HBOC-related cancers, including 50 (36.5%) with breast or ovarian cancer. Among the three females with cancer other than breast or ovarian, two had pancreatic cancer and one had melanoma. There were 3 (2.2%) variant-positive females who had more than one cancer, all of whom had both breast and ovarian cancers: one with *BRCA1* c.68_69delAG and two with *BRCA2* c.5946delT. Among variant-positive males (*N* = 81), 2 (2.5%) had breast cancer (*BRCA1* c.5266dupC and *BRCA2* c.4471_4474delCTGA) and 6 (7.4%) had prostate cancer (two men with *BRCA1* c.5266dupC and one man each with *BRCA1* c.68_69delAG, *BRCA2* c.2808_2811delACAA, *BRCA2* c.5946delT, and *BRCA2* c.4716_4717delinsAAAGACC). One of these men (1.2%) had more than one cancer (breast and pancreatic) and harbored *BRCA2* c.4471_4474delCTGA.

**Table 4:** Clinical characteristics of *BRCA1/2* variant-positive individuals. Evidence of HBOC-related cancers (breast, ovarian, prostate, pancreatic, and melanoma) and of clinical genetic testing among 218 Bio*Me* Biobank participants harboring expected pathogenic *BRCA1/2* variants

Population characteristics	Breast and ovarian cancers		All HBOC-related cancers		Evidence of clinical genetic testing, *N* (%)
	Personal history, *N* (%)	Personal or family history, *N* (%)	Personal history, *N* (%)	Personal or family history, *N* (%)	
All variant positive (*N* = 218)	52 (23.9)	88 (40.4)	61 (28.0)	98 (45.0)	58 (26.6)
By gene					
*BRCA1* (*N* = 86)	27 (31.4)	44 (51.2)	29 (33.7)	44 (51.2)	31 (36.0)
*BRCA2* (*N* = 131)	24 (18.3)	43 (32.8)	31 (23.7)	53 (40.5)	26 (19.8)
Both *BRCA1* and *BRCA2* (*N* = 1)	1 (100.0)	1 (100.0)	1 (100.0)	1 (100.0)	1 (100.0)
By gender					
Female (*N* = 137)	50 (36.5)	78 (56.9)	53 (38.7)	81 (59.1)	53 (38.7)
Male (*N* = 81)	2 (2.5)	10 (12.3)	8 (9.9)	17 (21.0)	5 (6.2)
*p* value (chi-squared test)	*3.9 × 10*^*−8*^	*2.3 × 10*^*−10*^	*9.7 × 10*^*−6*^	*9.9 × 10*^*−8 *^	*3.6 × 10*^*− 7*^
By founder variants					
With AJ founder variant (*N* = 80)	18 (22.5)	38 (47.5)	23 (28.8)	41 (51.3)	31 (38.8)
Without AJ founder variant (*N* = 138)	34 (24.6)	50 (36.2)	38 (27.5)	57 (41.3)	27 (19.6)
*p* value (chi-squared test)	*0.85*	*0.14*	*0.97*	*0.18*	*3.4 × 10*^*−3*^

We assessed the number of variant-positive individuals with prior knowledge of their *BRCA1/2* variant status. Review of medical records revealed that 58 (26.6%) had EHR evidence of clinical genetic testing for *BRCA1/2* (Table 4). Among 98 variant-positive individuals with a personal or family history of HBOC-related cancer, 51 (52.0%) had evidence of clinical genetic testing. Only 5 of 81 (6.2%) males had evidence of clinical genetic testing, compared with 53 of 137 (38.7%) females (chi-squared *p* = 3.6 × 10^− 7^). Although personal rates of cancer were similar among individuals with AJ founder variants and those with other variants (28.8% vs. 27.5%, chi-squared *p* = 0.97), knowledge of *BRCA1/2* variant status varied: 31 of 80 (38.8%) individuals with AJ founder variants had documented evidence of clinical genetic testing, compared with only 27 of 138 (19.6%) individuals harboring other *BRCA1/2* variants (chi-squared *p* = 3.4 × 10^− 3^).

We tested for association with HBOC-related cancers in variant-positive (*N* = 208) compared with variant-negative (not harboring any ClinVar pathogenic, uncertain/conflicting, or novel variants; *N* = 24,927) participants in the unrelated subset. Variant-positive individuals had increased odds of HBOC-related cancers (odds ratio (OR) 5.6; 95% confidence interval (CI) 4.0 to 8.0; *p* = 6.7 × 10^− 23^). In contrast, participants harboring uncertain/conflicting variants (*N* = 2395) did not have increased odds of HBOC-related cancers (OR 1.2; 95% CI 1.0 to 1.4; *p* = 0.1). To more comprehensively evaluate the clinical consequences of expected pathogenic variants in *BRCA1/2*, we performed a PheWAS of variant-positive vs. variant-negative participants. Using a Bonferroni significance threshold of *p* = 1.9 × 10^− 4^ for associations with 260 clinical diagnoses, we identified significant associations with “malignant neoplasm of female breast” (OR 8.1; 95% CI 5.4 to 12.2; *p* = 2.2 × 10^− 23^) and “other specified disorders of breast” (OR 6.9; 95% CI 2.9 to 16.2; *p* = 9.0 × 10^− 6^; Additional file 1: Figure S2). There were no associations with other types of cancer or non-cancer phenotypes, including known HBOC-related cancers, suggesting we may have been underpowered to observe other relevant associations.

## Discussion

In this study, we demonstrate the ability of large-scale, population-based genomic sequencing to identify and characterize consequential variants in *BRCA1/2* in a large, ethnically diverse health system. We found an overall prevalence of 1 in 139 individuals with expected pathogenic variants in *BRCA1/2*, observed differing frequencies of such variants among a broad range of represented ancestries, and discovered that the majority of individuals harboring these variants were unaware of their genomic risk status.

The overall prevalence of expected pathogenic *BRCA1/2* variants in our population was higher than previous estimates [5, 6, 13] and may be partly explained by the large number of founder variants detected. The highest prevalence was 1 in 49 (2.1%) in individuals with AJ genetic ancestry, which is similar to the previously established prevalence of 1 in 42 (2.4%) in this population [7, 8]. The high proportion of AJ individuals in our cohort (14.0%) contributed to the high overall prevalence observed. Multiple other founder variants were also detected in different populations in our study, including the c.3922G>T (p.Glu1308Ter) variant in *BRCA2* that we found in almost half of the variant-positive individuals with ancestry from PR, consistent with previous findings [47]. We report, for the first time, prevalence estimates in a number of diverse populations, including African American and Hispanic/Latino populations for which these estimates did not previously exist.

Our findings also revealed that non-European populations, and particularly those most genetically divergent from European populations, are more likely to harbor *BRCA1/2* variants that are not classified in public databases or that have uncertain or conflicting evidence for pathogenicity. This was also evident in mixed ancestry populations such as Hispanic/Latino populations, in whom the proportion of variants with uncertain/conflicting interpretations correlated with the percent African genetic ancestry. While *BRCA1/2* variant-positive individuals had significantly increased risk of HBOC-related cancers, those with uncertain/conflicting variants did not, suggesting that many of these variants are likely to be benign or of low penetrance. These data add to a growing body of literature [19–21] underscoring the pressing need to further characterize genomic variation across diverse populations.

As with previous studies, there was a higher rate of relevant cancers in *BRCA1* variant-positive individuals than in *BRCA2*, and in women than in men [13, 54, 55]. Over one-third of the variant-positive females in our study had a documented current or prior diagnosis of a HBOC-related cancer. Genomic screening in individuals with cancer still provides an opportunity for early detection or prophylaxis, as evidenced by the finding of a second primary cancer in four participants. Genomic screening in apparently healthy men may represent an opportunity for intervention through increased prostate surveillance, given the recently recognized contribution of germline *BRCA1*/2 variants to metastatic prostate cancer burden [56].

Knowledge of *BRCA1/2* status as documented in participant EHRs was only 27% overall, and even lower (20%) in individuals with non-AJ founder variants, confirming prior reports of clinical under-ascertainment [13]. Of note, 10% of the variant-positive AJ individuals harbored non-founder variants, consistent with previous findings [57] and highlighting the need for comprehensive testing of *BRCA1/2* genes rather than targeted screening for specific founder variants in this population. The observed difference in clinical testing among individuals with or without AJ founder variants, despite similar rates of cancer, indicates that there may be additional barriers to genetic testing in populations that are not considered higher risk on the basis of ancestry. Obstacles in non-AJ populations could include lack of patient awareness about *BRCA1/2*, lower suspicion for HBOC by healthcare providers, or reduced access and/or uptake of genetic testing in certain populations within the context of broader healthcare disparities. Such barriers have been described in African American and Hispanic/Latino populations, the two largest non-European populations in Bio*Me*, suggesting that interventions to improve awareness, risk perception, and patient-provider communication are needed to reduce disparities in *BRCA1/2* testing in diverse populations [58].

Current evidence- and expert opinion-driven guidelines [10, 11, 59] as well as statistical models [60–63] to identify potential candidates for *BRCA1/2* testing are mainly based on the number of individuals with relevant cancers in a kindred, age(s) of diagnosis, and ancestry. Testing criteria have widened over time with the recognition that they do not sufficiently identify all individuals harboring a *BRCA1/2* pathogenic variant. Nevertheless, our findings suggest that current clinical practices still miss a significant opportunity for reducing morbidity and mortality through identification of high-risk variant-positive individuals. While we were unable to evaluate whether variant-positive individuals would meet current testing criteria, we did observe that almost half of those with a relevant personal or family history of cancer had no evidence of clinical *BRCA1/2* testing. The potential for improved health outcomes from genomic screening through ascertainment of patients and identification of at-risk relatives through cascade testing [64, 65] supports the Centers for Disease Control and Prevention’s designation of HBOC as a tier 1 genomic condition for which positive public health impact exists (https://www.cdc.gov/genomics/implementation/toolkit/tier1.htm).

There are limitations to our study. The study population consisted of individuals recruited from clinical care sites, which does not necessarily reflect the general population of New York City. However, these findings do provide insight into diverse patient populations that were ascertained in a relatively unselected, population-based manner and that have not been previously represented in similar research efforts. The observed prevalence of *BRCA1/2* expected pathogenic variants may represent an underestimate, as certain variants would not be detected via this approach, including large copy number variants, which make up approximately 10% of all *BRCA1/2* pathogenic variants [66–69]. Additionally, some percentage of variants of uncertain significance may in fact be pathogenic and likely will be classified as such in the future. We were also constrained by the use of EHR-extracted clinical information, which may not reflect complete medical and family history [70], and may downwardly bias the true penetrance of HBOC in our cohort.

## Conclusions

Genomic screening for pathogenic *BRCA1/2* variants in apparently healthy individuals has the potential to lead to earlier diagnosis of cancer via increased surveillance, as well as cancer risk reduction via prophylactic medical interventions. In this study, we provide evidence for a higher overall prevalence of *BRCA1/2* expected pathogenic variants in the Bio*Me* Biobank than historically appreciated, in line with recent findings from another unselected clinical care cohort [13]. We show that this approach can effectively identify at-risk individuals across ethnically diverse and underserved populations such as those present in Bio*Me*. These findings are in part due to the cross-sectional representation of founder variants from multiple different populations, which accounted for over half of individuals harboring pathogenic variants in this study. We demonstrate that genomic screening for *BRCA1/2* in diverse patient populations may be an effective tool to identify otherwise unrecognized HBOC-associated variants, in order to prevent or diagnose disease. However, further work is needed to accurately classify pathogenic variants in non-European populations, in order to most effectively use this strategy to improve health outcomes in diverse settings.

## Supplementary information

**Additional file 1: Figure S1.** Correlation between proportion African genetic ancestry and the likelihood of harboring an uncertain/conflicting *BRCA1/2* variant in Hispanic/Latinos. **Figure S2.** Phenome-wide association study of *BRCA1/2* variant-positive vs. variant-negative participants using EHR-derived clinical diagnoses (phecodes). **Table S1.** International Classification of Diseases (ICD)-9 and − 10 codes used to characterize *BRCA1/2* variant-positive individuals. **Table S2.** Distribution of 1601 *BRCA1/2* variants obtained from exome sequence data available from 30,223 adult Bio*Me* Biobank participants, according to ClinVar assertion and variant type. **Table S3.** *BRCA1/2* expected pathogenic variants identified in 30,223 exome sequenced adults from the Bio*Me* Biobank.

**Additional file 2.** Banner Author Lists and Contribution Statements. The Charles Bronfman Institute of Personalized Medicine (CBIPM) Genomics Team Banner Author List and Contribution Statements. Regeneron Genetics Center Banner Author List and Contribution Statements.

## Data Availability

Expected pathogenic variants in BRCA1/2 reported in this paper are tabulated in Additional file 1: Table S3. Summary statistics, including genotype counts across self-reported and genetic ancestry groups from Bio*Me*, for all *BRCA1/2* variants are available at https://sinaigenomichealth.org/research-resources/. Exome sequencing and genotyping of Bio*Me* was performed in collaboration with the Regeneron Genetics Center. Individual-level data generated via this collaboration are not publicly available due to the terms of the Bio*Me* biospecimen and data access agreement but may be requested directly from the corresponding author.
