# Supplementary material for: Exome sequencing reveals a high prevalence of BRCA1 and BRCA2 founder variants in a diverse population-based biobank
Source: Genome Med. 2019 Dec 31;12:2. doi: 10.1186/s13073-019-0691-1 (PMC6938627; doi:10.1186/s13073-019-0691-1)
Supplement: Supplementary file 1 — Additional file 1: Figure S1. Correlation between proportion African genetic ancestry and the likelihood of harboring an uncertain/conflicting BRCA1/2 variant in Hispanic/Latinos. Figure S2. Phenome-wide association study of BRCA1/2 variant-positive vs. variant-negative participants using EHR-derived clinical diagnoses (phecodes). Table S1. International Classification of Diseases (ICD)-9 and − 10 codes used to characterize BRCA1/2 variant-positive individuals. Table S2. Distribution of 1601 BRCA1/2 variants obtained from exome sequence data available from 30,223 adult BioMe Biobank participants, according to ClinVar assertion and variant type. Table S3. BRCA1/2 expected pathogenic variants identified in 30,223 exome sequenced adults from the BioMe Biobank. [file 13073_2019_691_MOESM1_ESM.pdf]

ADDITIONAL FILE 1

Exome Sequencing Reveals a High Prevalence of *BRCA1* and *BRCA2* Founder Variants in a Diverse Population-Based Biobank

Noura S. Abul-Husn[1,2,3,4*], Emily R. Soper[1,2†], Jacqueline A. Odgis[1,2†], Sinead Cullina[1,2], Dean Bobo[1,2], Arden Moscati[1,2], Jessica E. Rodriguez[1,2], CBIPM Genomics team[1,2], Regeneron Genetics Center[5], Ruth J.F. Loos[2], Judy H. Cho[2,3,4], Gillian M. Belbin[1,2,3], Sabrina A. Suckiel[1,2], Eimear E. Kenny[1,2,3,4]

[1]The Center for Genomic Health, Icahn School of Medicine at Mount Sinai, New York, NY
[2]The Charles Bronfman Institute for Personalized Medicine, Icahn School of Medicine at Mount Sinai, New York, NY
[3]Department of Medicine, Icahn School of Medicine at Mount Sinai, New York, NY
[4]Department of Genetics and Genomic Sciences, Icahn School of Medicine at Mount Sinai, New York, NY
[5]Regeneron Genetics Center, Tarrytown, New York, NY

[†] These authors contributed equally to this work

***Correspondence:** noura.abul-husn@mssm.edu

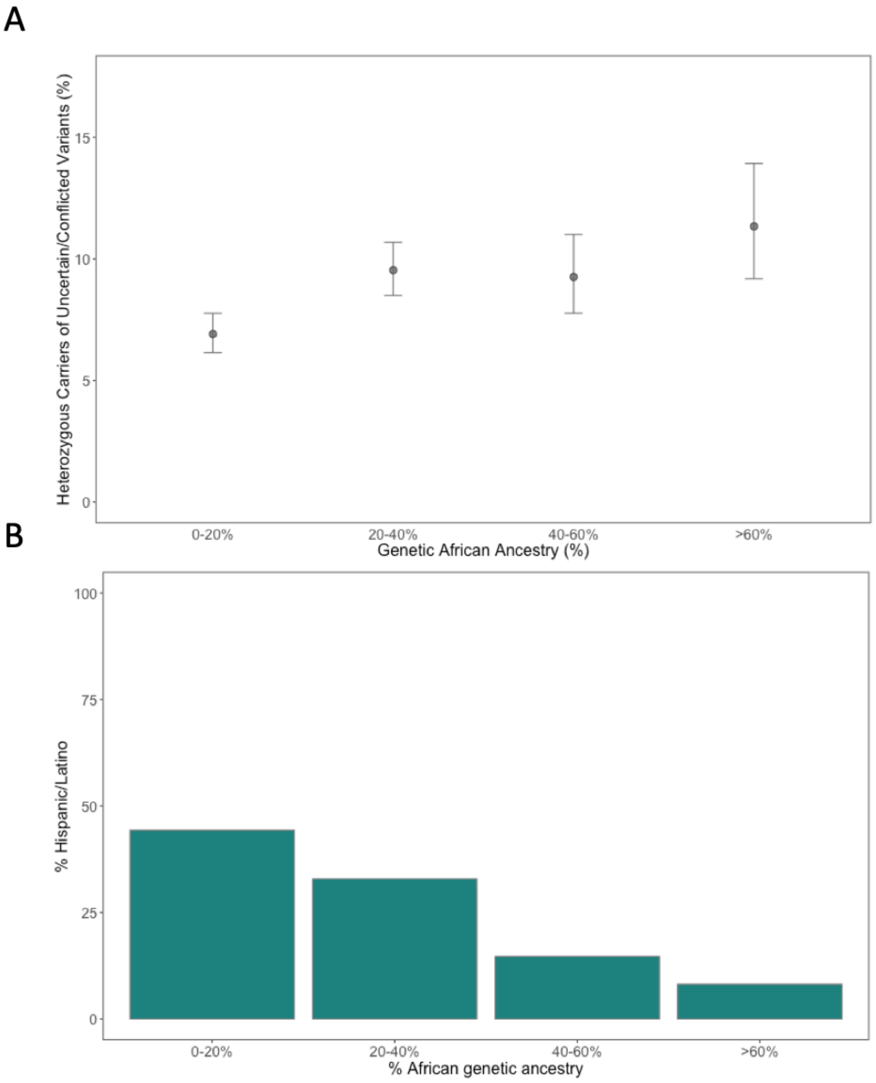

Figure S1. Correlation between proportion African genetic ancestry and the likelihood of harboring an uncertain/conflicting *BRCA1/2* variant in Hispanic/Latinos. Among 394 *BRCA1/2* variants identified in unrelated, self-reported Hispanic/Latinos with both exome sequence and GSA genotype data in the Bio*Me* Biobank (*N* = 8457), there were 125 variants of uncertain significance or with conflicting interpretations of pathogenicity in ClinVar (uncertain/conflicting). We examined the proportion of uncertain/conflicting variants in four bins of African genetic ancestry (0-20%, 20-40%, 40-60%, 60-100%) and found a strong correlation (Pearson correlation coefficient 0.96) between higher proportion African genetic ancestry and higher rates of uncertain/conflicting variants (**1A**). Hispanic/Latino participants in the highest African genetic ancestry bin had an almost twofold higher rate of uncertain/conflicting variants (11.3%, 95% CI 9.2-13.9%) compared to those in the lowest bin (6.9%, 95% CI 6.1-7.7%); chi-squared test p = 7.8×10^{-5}. **1B** shows the breakdown of self-reported Hispanic/Latinos by African genetic ancestry bins; 0-20% (*N* = 3748); >20-40% (*N* = 2779); >40-60% (*N* = 1242); >60% (*N* = 688).

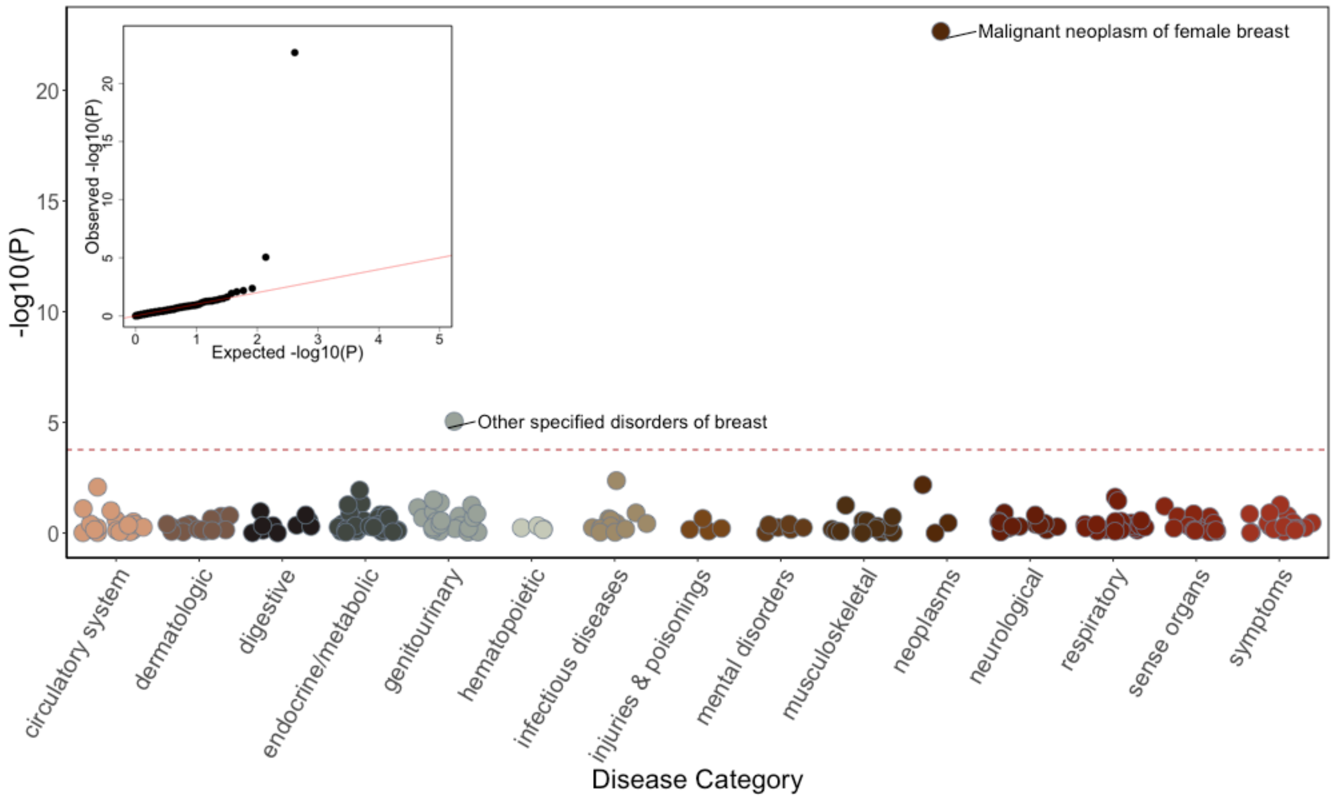

Figure S2. Phenome-wide association study of *BRCA1/2* variant-positive vs. variant-negative participants using EHR-derived clinical diagnoses (phecodes). We restricted analyses to phecodes present in at least 5 variant-positive participants, resulting in a total of 260 tests. Logistic regression was performed adjusting for age, sex, and the first 5 principal components. Using a Bonferroni adjusted significance threshold of $p < 1.9 \times 10^{-4}$, we identified two significant associations of variant-positive participants with “Malignant neoplasm of female breast” (OR 8.1; 95% CI 5.4 to 12.2; $p = 2.2 \times 10^{-23}$) and “Other specified disorders of breast” (OR 6.9; 95% CI 2.9 to 16.2; $p = 9.0 \times 10^{-6}$).

Table S1. International Classification of Diseases (ICD)-9 and -10 codes used to characterize *BRCA1/2* variant-positive individuals.

Personal and Family Medical History		ICD-9 Codes	ICD-10 Codes	Definition
**Personal History**	Breast cancer	174.*, 175.*, 233.0, V10.3	C50.*, D05.*, Z85.3, Z86.000	Current or past diagnosis of invasive and/or in situ carcinoma of the breast (male or female)
	Ovarian cancer	183.*, V10.43	C56.*, C57.0*, Z85.43	Current or past diagnosis of carcinoma of the ovary
	Prostate cancer	185.*, 233.4, V10.46	C61, Z85.46, D07.5	Current or past diagnosis of carcinoma of the prostate
	Pancreatic cancer	157.*	C25.*, Z85.07	Current or past diagnosis of carcinoma of the pancreas
	Melanoma	172.*, V10.82	C43*, Z85.820	Current or past diagnosis of cutaneous melanoma
	Prior Genetic Testing	V84.01, V84.02, V84.03	Z15.01, Z15.02, Z15.03	Genetic susceptibility to breast, ovary or prostate
**Family History**	Breast cancer	V16.3	Z80.3	Family history of breast cancer
	Ovarian cancer	V16.41	Z80.41	Family history of ovarian cancer
	Prostate cancer	V16.42	Z80.42	Family history of prostate cancer
	Gastrointestinal tract cancer	V16.0	Z80.0	Family history of cancer of the gastrointestinal tract (including pancreas)
	Prior Genetic Testing	V18.9	Z84.81	Family history of genetic disease

Table S2. Distribution of 1601 *BRCA1/2* variants obtained from exome sequence data available from 30,223 adult Bio*Me* Biobank participants, according to ClinVar assertion and variant type.

**ClinVar Assertion**	***BRCA1*, N (%)**	***BRCA2*, N (%)**	***BRCA1/2*, N (%)**
Pathogenic/Likely Pathogenic	31 (5.6)	71 (6.8)	102 (6.4)
Uncertain Significance	174 (31.4)	356 (34.0)	530 (33.1)
Conflicting Interpretations	27 (4.9)	78 (7.5)	105 (6.6)
Benign/Likely Benign	238 (42.9)	360 (34.4)	598 (37.4)
NA	85 (15.3)	181 (17.3)	266 (16.6)*
**Variant Type[#]**			
Frameshift	12 (2.2)	44 (4.2)	56 (3.5)
Missense	361 (65.0)	655 (62.6)	1016 (63.5)
Stop gained	12 (2.2)	19 (1.8)	31 (1.9)
Synonymous	150 (27.0)	221 (21.1)	371 (23.2)
NA	20 (3.6)	107 (10.2)	127 (7.9)
**Total**	**555**	**1046**	**1601**

*10 of the 266 variants with no ClinVar assertions were predicted loss-of-function variants (frameshift or stop gained) and were included in the set of expected pathogenic variants used to define variant-positive individuals.

[#]Consequence based on Variant Effect Predictor annotation.

Table S3. *BRCA1/2* expected pathogenic variants identified in 30,223 exome sequenced adults from the Bio*Me* Biobank.

CHR:POS:REF:ALT	Gene	Function	rsID	cDNA Position	Protein Position	ClinVar Significance (Review Status)	# Hets	Self-Reported Ancestry (# Hets)	Genetic Ancestry (# Hets)
17:43049191:TG:T	*BRCA1*	Frameshift	rs80357590	c.5335delC	p.Gln1779fs	Pathogenic (3*)	1	ESA (1)	Filipino and other Southeast Asian (1)
17:43051071:A:C	*BRCA1*	Missense	rs41293463	c.5324T>G	p.Met1775Arg	Pathogenic (3*)	3	AA (3)	African American and African (3)
17:43057062:T:TG	*BRCA1*	Frameshift	rs80357906	c.5266dupC	p.Gln1756fs	Pathogenic (3*)	6	EA (6)	AJ (5), Non-AJ European (1)
17:43063345:TTTTC:T	*BRCA1*	Frameshift	rs80357867	c.5177_5180delGAAA	p.Arg1726fs	Pathogenic (3*)	1	AA (1)	
17:43063903:G:T	*BRCA1*	Missense	rs28897696	c.5123C>A	p.Ala1708Glu	Pathogenic (3*)	1	EA (1)	Non-AJ European (1)
17:43063917:A:C	*BRCA1*	Stop gained	rs80356974	c.5109T>G	p.Tyr1703Ter	Pathogenic (3*)	1	EA (1)	Non-AJ European (1)
17:43071017:TC:T	*BRCA1*	Frameshift		c.4896delG	p.Ser1633fs	NA	1	EA (1)	Non-AJ European (1)
17:43074403:C:A	*BRCA1*	Stop gained	rs80357366	c.4603G>T	p.Glu1535Ter	Pathogenic (3*)	3	AA (3)	African American and African (2)
17:43076559:GC:G	*BRCA1*	Frameshift	rs1064793951	c.4412delG	p.Gly1471fs	Pathogenic (3*)	1	HA (1)	Colombian (2)
17:43082434:G:A	*BRCA1*	Stop gained	rs41293455	c.4327C>T	p.Arg1443Ter	Pathogenic (3*)	1	O (1)	Non-AJ European (1)
17:43091443:G:C	*BRCA1*	Stop gained	rs398122680	c.4088C>G	p.Ser1363Ter	Pathogenic (3*)	1	AA (1)	African American and African (1)
17:43091455:T:TGC	*BRCA1*	Frameshift		c.4074_4075dupGC	p.Gln1359fs	NA	1	ESA (1)	Filipino and other Southeast Asian (1)
17:43091669:CCTCA:C	*BRCA1*	Frameshift	rs80357842	c.3858_3861delTGAG	p.Ser1286fs	Pathogenic (3*)	1	HA (1)	Central and South American (1)
17:43091714:G:A	*BRCA1*	Stop gained	rs80357208	c.3817C>T	p.Gln1273Ter	Pathogenic (3*)	1	HA (1)	Puerto Rican (1)
17:43091771:TAGAC:T	*BRCA1*	Frameshift	rs80357868	c.3756_3759delGTCT	p.Ser1253fs	Pathogenic (3*)	2	EA (2)	Non-AJ European (1)
17:43091924:G:A	*BRCA1*	Stop gained	rs62625308	c.3607C>T	p.Arg1203Ter	Pathogenic (3*)	2	EA (1), HA (1)	Puerto Rican (1)
17:43092119:C:A	*BRCA1*	Stop gained	rs886040126	c.3412G>T	p.Gly1138Ter	Pathogenic (3*)	1	AA (1)	African American and African (1)
17:43092196:TCTTG:T	*BRCA1*	Frameshift	rs80357701	c.3331_3334delCAAG	p.Gln1111fs	Pathogenic (3*)	2	AA (1), HA (1)	African American and African (1), Central and South American (1)
17:43092570:C:CT	*BRCA1*	Frameshift	rs886040088	c.2960dupA	p.Ser988fs	Pathogenic (3*)	1	EA (1)	Non-AJ European (1)
17:43092615:TC:T	*BRCA1*	Frameshift	rs80357573	c.2915delG	p.Gly972fs	Pathogenic (3*)	1	AA (1)	African American and African (1)
17:43093055:TG:T	*BRCA1*	Frameshift	rs80357970	c.2475delC	p.Asp825fs	Pathogenic (3*)	1	EA (1)	AJ (1)
17:43093496:T:A	*BRCA1*	Stop gained	rs80357082	c.2035A>T	p.Lys679Ter	Pathogenic (3*)	1	EA (1)	Non-AJ European (1)
17:43093950:CTT:C	*BRCA1*	Frameshift	rs431825387	c.1579_1580delAA	p.Lys527fs	Pathogenic (3*)	1	EA (1)	Non-AJ European (1)
17:43094018:T:A	*BRCA1*	Stop gained	rs397508877	c.1513A>T	p.Lys505Ter	Pathogenic (3*)	1	HA (1)	
17:43094451:G:T	*BRCA1*	Stop gained	rs886037986	c.1080C>A	p.Cys360Ter	Pathogenic (3*)	1	EA (1)	Non-AJ European (1)
17:43094472:C:T	*BRCA1*	Stop gained	rs80356935	c.1059G>A	p.Trp353Ter	Pathogenic (3*)	1	O (1)	Non-AJ European (1)
17:43104260:A:C	*BRCA1*	Stop gained	rs80356936	c.303T>G	p.Tyr101Ter	Pathogenic (3*)	1	AA (1)	African American and African (1)

CHR:POS:REF:ALT	Gene	Function	rsID	cDNA Position	Protein Position	ClinVar Significance (Review Status)	# Hets	Self-Reported Ancestry (# Hets)	Genetic Ancestry (# Hets)
17:43106457:T:C	*BRCA1*	Missense	rs80357382	c.211A>G	p.Arg71Gly	Pathogenic (2*)	3	HA (2), ESA (1)	Puerto Rican (2), Filipino and other Southeast Asian (1)
17:43106478:A:C	*BRCA1*	Missense	rs80357064	c.190T>G	p.Cys64Gly	Pathogenic (2*)	1	AA (1)	African American and African (1)
17:43106487:A:C	*BRCA1*	Missense	rs28897672	c.181T>G	p.Cys61Gly	Pathogenic (3*)	1	EA (1)	Non-AJ European (1)
17:43115744:C:T	*BRCA1*	Missense	rs80357498	c.116G>A	p.Cys39Tyr	Pathogenic (2*)	1	M (1)	
17:43124027:ACT:A	*BRCA1*	Frameshift	rs80357914	c.68_69delAG	p.Glu23fs	Pathogenic (3*)	41	EA (36), M (4), O (1)	AJ (38)
17:43124030:C:CT	*BRCA1*	Frameshift	rs80357783	c.66dupA	p.Glu23fs	Pathogenic (3*)	1	O (1)	Non-AJ European (1)
13:32319298:G:T	*BRCA2*	Stop gained	rs397507646	c.289G>T	p.Glu97Ter	Pathogenic (3*)	1	O (1)	Non-AJ European (1)
13:32329467:CTG:C	*BRCA2*	Frameshift	rs768580992	c.658_659delGT	p.Val220fs	Pathogenic (3*)	1	EA (1)	Non-AJ European (1)
13:32330986:TGACA:T	*BRCA2*	Frameshift	rs80359659	c.755_758delACAG	p.Asp252fs	Pathogenic (3*)	1	EA (1)	Non-AJ European (1)
13:32331009:C:T	*BRCA2*	Stop gained	rs80358998	c.772C>T	p.Gln258Ter	Pathogenic (3*)	2	EA (2)	Non-AJ European (2)
13:32332517:C:T	*BRCA2*	Stop gained		c.1039C>T	p.Gln347Ter	NA	1	AA (1)	African American and African (1)
13:32332778:AAAAG:A	*BRCA2*	Frameshift	rs80359277	c.1310_1313delAAGA	p.Lys437fs	Pathogenic (3*)	1	HA (1)	Ecuadorian (1)
13:32332862:G:T	*BRCA2*	Stop gained		c.1384G>T	p.Glu462Ter	NA	1	AA (1)	
13:32333165:TG:T	*BRCA2*	Frameshift	rs876659278	c.1689delG	p.Trp563fs	Pathogenic (3*)	1	EA (1)	Non-AJ European (1)
13:32333228:AAAAAG:A	*BRCA2*	Frameshift	rs80359302	c.1755_1759delGAAAA	p.Lys585fs	Pathogenic (3*)	1	EA (1)	Non-AJ European (1)
13:32333282:G:T	*BRCA2*	Stop gained		c.1804G>T	p.Gly602Ter	NA	1	HA (1)	Puerto Rican (1)
13:32333363:CTTACATT:C	*BRCA2*	Frameshift	rs1555282146	c.1887_1893delTACATTT	p.Thr630fs	Pathogenic (3*)	1	AA (1)	African American and African (1)
13:32336684:G:GA	*BRCA2*	Frameshift	rs80359328	c.2330dupA	p.Asp777fs	Pathogenic (3*)	1	EA (1)	Non-AJ European (1)
13:32336750:A:T	*BRCA2*	Stop gained		c.2395A>T	p.Lys799Ter	NA	1	EA (1)	AJ (1)
13:32336781:T:G	*BRCA2*	Stop gained	rs397507285	c.2426T>G	p.Leu809Ter	Pathogenic (3*)	1	EA (1)	Non-AJ European (1)
13:32337055:TC:T	*BRCA2*	Frameshift	rs397507637	c.2701delC	p.Ala902fs	Pathogenic (3*)	1	HA (1)	Puerto Rican (1)
13:32337160:TA:T	*BRCA2*	Frameshift	rs398122753	c.2808del	p.Lys936fs	Pathogenic (3*)	1	AA (1)	African American and African (1)
13:32337160:TAAAC:T	*BRCA2*	Frameshift	rs80359351	c.2808_2811delACAA	p.Ala938fs	Pathogenic (3*)	1	HA (1)	Central and South American (1)
13:32337305:G:GA	*BRCA2*	Frameshift	rs80359365	c.2957dupA	p.Asn986fs	Pathogenic (3*)	1	EA (1)	Non-AJ European (1)
13:32337312:A:AG	*BRCA2*	Frameshift	rs1555282969	c.2957_2958insG	p.Asn986fs	Pathogenic (3*)	1	AA (1)	African American and African (1)
13:32337952:CTG:C	*BRCA2*	Stop gained	rs80359391	c.3599_3600delGT	p.Cys1200Terfs	Pathogenic (3*)	1	EA (1)	Non-AJ European (1)
13:32338034:CTG:C	*BRCA2*	Frameshift	rs80359395	c.3680_3681delTG	p.Leu1227fs	Pathogenic (3*)	1	AA (1)	African American and African (1)
13:32338069:GA:G	*BRCA2*	Frameshift	rs80359401	c.3717delA	p.Lys1239fs	Pathogenic (3*)	1	EA (1)	Non-AJ European (1)
13:32338075:GT:G	*BRCA2*	Frameshift	NA	c.3723delT	p.Phe1241fs	Pathogenic (3*)	1	EA (1)	AJ (1)

CHR:POS:REF:ALT	Gene	Function	rsID	cDNA Position	Protein Position	ClinVar Significance (Review Status)	# Hets	Self-Reported Ancestry (# Hets)	Genetic Ancestry (# Hets)
13:32338200:CTG:C	*BRCA2*	Frameshift	rs80359405	c.3847_3848delGT	p.Val1283fs	Pathogenic (3*)	3	EA (2), M (1)	Non-AJ European (2), African American and African (1)
13:32338212:AAAAT:A	*BRCA2*	Frameshift	rs80359410	c.3860_3863delATAA	p.Asn1287fs	Pathogenic (3*)	1	O (1)	African American and African (1)
13:32338277:G:T	*BRCA2*	Stop gained	rs80358638	c.3922G>T	p.Glu1308Ter	Pathogenic (3*)	8	HA (8)	Puerto Rican (7)
13:32338565:TC:T	*BRCA2*	Stop gained	rs398122777	c.4211delC	p.Ser1404Terfs	Pathogenic (3*)	1	AA (1)	African American and African (1)
13:32338783:CA:C	*BRCA2*	Frameshift	rs886040531	c.4429delA	p.Ile1477fs	Pathogenic (3*)	1	EA (1)	Non-AJ European (1)
13:32338824:TACTG:T	*BRCA2*	Frameshift	rs80359451	c.4471_4474delCTGA	p.Leu1491fs	Pathogenic (3*)	1	AA (1)	African American and African (1)
13:32338968:CT:C	*BRCA2*	Frameshift		c.4616delT	p.Leu1539fs	NA	1	HA (1)	Central and South American (1)
13:32338981:GA:G	*BRCA2*	Frameshift	rs80359461	c.4631delA	p.Asn1544fs	Pathogenic (3*)	1	O (1)	Filipino and other Southeast Asian (1)
13:32338986:A:AG	*BRCA2*	Frameshift		c.4631_4632insG	p.Asn1544fs	NA	1	AA (1)	
13:32339069:CCTG:GCAAAGACC	*BRCA2*	Frameshift		c.4716_4717delinsAAAGACC	p.Cys1573fs	NA	1	O (1)	Non-AJ European (1)
13:32339084:GA:G	*BRCA2*	Frameshift	rs397507740	c.4731delA	p.Glu1577fs	Pathogenic (3*)	1	AA (1)	African American and African (1)
13:32339318:T:TA	*BRCA2*	Frameshift	rs398122789	c.4964dupA	p.Tyr1655fs	Pathogenic (3*)	1	EA (1)	Non-AJ European (1)
13:32339320:C:G	*BRCA2*	Stop gained	rs80358721	c.4965C>G	p.Tyr1655Ter	Pathogenic (3*)	1	M (1)	
13:32339394:CTG:C	*BRCA2*	Frameshift	rs80359478	c.5042_5043delTG	p.Val1681fs	Pathogenic (3*)	1	EA (1)	Non-AJ European (1)
13:32339421:C:CA	*BRCA2*	Frameshift	rs80359479	c.5073dupA	p.Trp1692fs	Pathogenic (3*)	1	EA (1)	Non-AJ European (1)
13:32339421:CA:C	*BRCA2*	Frameshift	rs80359479	c.5073delA	p.Lys1691fs	Pathogenic (3*)	1	HA (1)	Ecuadorian (1)
13:32339460:CAGAA:C	*BRCA2*	Frameshift	NA	c.5110_5113delAGAA	p.Arg1704fs	Pathogenic (3*)	1	EA (1)	Non-AJ European (1)
13:32339489:G:T	*BRCA2*	Stop gained	NA	c.5134G>T	p.Gly1712Ter	Pathogenic (3*)	1	EA (1)	Non-AJ European (1)
13:32339571:ATTTAAGT:A	*BRCA2*	Frameshift	rs80359496	c.5217_5223delTTTAAGT	p.Tyr1739fs	Pathogenic (3*)	1	EA (1)	Non-AJ European (1)
13:32339699:C:CA	*BRCA2*	Frameshift	rs80359507	c.5351dupA	p.Asn1784fs	Pathogenic (3*)	1	M (1)	Non-AJ European (1)
13:32339699:CAA:C	*BRCA2*	Frameshift	rs80359507	c.5350_5351delAA	p.Asn1784fs	Pathogenic (3*)	1	EA (1)	Non-AJ European (1)
13:32339928:CAATT:C	*BRCA2*	Frameshift	rs770318608	c.5576_5579delTTAA	p.Ile1859fs	Pathogenic (3*)	1	ESA (1)	Filipino and other Southeast Asian (1)
13:32340128:C:T	*BRCA2*	Stop gained	rs80358806	c.5773C>T	p.Gln1925Ter	Pathogenic (3*)	1	EA (1)	AJ (1)
13:32340149:CAT:C	*BRCA2*	Frameshift	rs763890036	c.5796_5797delTA	p.His1932fs	Pathogenic (3*)	1	EA (1)	Non-AJ European (1)
13:32340151:TAACC:T	*BRCA2*	Frameshift	rs80359538	c.5799_5802delCCAA	p.Asn1933fs	Pathogenic (3*)	1	HA (1)	Puerto Rican (1)
13:32340202:TGTTA:T	*BRCA2*	Frameshift	rs80359543	c.5851_5854delAGTT	p.Ser1951fs	Pathogenic (3*)	2	HA (2)	Dominican (2)
13:32340212:G:T	*BRCA2*	Stop gained	rs80358814	c.5857G>T	p.Glu1953Ter	Pathogenic (3*)	1	AA (1)	African American and African (1)
13:32340300:GT:G	*BRCA2*	Frameshift	rs80359550	c.5946delT	p.Ser1982fs	Pathogenic (3*)	34	EA (30), M (1), NA (1), O (2)	AJ (30), Non-AJ European (1)

CHR:POS:REF:ALT	Gene	Function	rsID	cDNA Position	Protein Position	ClinVar Significance (Review Status)	# Hets	Self-Reported Ancestry (# Hets)	Genetic Ancestry (# Hets)
13:32340392:A:T	*BRCA2*	Stop gained	rs80358840	c.6037A>T	p.Lys2013Ter	Pathogenic (3*)	2	EA (2)	Non-AJ European (2)
13:32340629:CTT:C	*BRCA2*	Frameshift	rs11571658	c.6275_6276delTT	p.Leu2092fs	Pathogenic (3*)	1	EA (1)	
13:32340751:A:AT	*BRCA2*	Frameshift	rs431825342	c.6397dupT	p.Ser2133fs	Pathogenic (3*)	1	O (1)	Non-AJ European (1)
13:32340816:ATC:A	*BRCA2*	Frameshift	rs80359596	c.6468_6469delTC	p.Gln2157fs	Pathogenic (3*)	2	HA (1), O (1)	Non-AJ European (1)
13:32340836:GACAA:G	*BRCA2*	Frameshift	rs770263702	c.6486_6489delACAA	p.Lys2162fs	Pathogenic (3*)	1	HA (1)	Central and South American (1)
13:32340882:ACATT:A	*BRCA2*	Frameshift	rs397507865	c.6531_6534delTCAT	p.Ile2177fs	Pathogenic (3*)	1	HA (1)	Central and South American (1)
13:32340983:GAA:G	*BRCA2*	Frameshift	rs80359611	c.6629_6630delAA	p.Glu2210fs	Pathogenic (3*)	1	HA (1)	Central and South American (1)
13:32340998:TACTC:T	*BRCA2*	Frameshift	rs80359616	c.6644_6647delACTC	p.Tyr2215fs	Pathogenic (3*)	1	HA (1)	African American and African (1)
13:32341035:C:CA	*BRCA2*	Frameshift	rs1555284790	c.6681dupA	p.Val2228fs	Pathogenic (3*)	1	HA (1)	Puerto Rican (1)
13:32354920:TTC:T	*BRCA2*	Frameshift	rs756538291	c.7069_7070delCT	p.Leu2357fs	Pathogenic (3*)	1	O (1)	Non-AJ European (1)
13:32355068:C:CT	*BRCA2*	Frameshift	rs876659345	c.7218dupT	p.Val2407fs	Pathogenic (3*)	1	EA (1)	AJ (1)
13:32355212:GA:G	*BRCA2*	Frameshift	rs80359646	c.7360delA	p.Ile2454fs	Pathogenic (3*)	1	EA (1)	Non-AJ European (1)
13:32355270:T:TTC	*BRCA2*	Frameshift		c.7417_7418insTC	p.Cys2473fs	NA	1	ESA (1)	
13:32356463:C:T	*BRCA2*	Stop gained	rs80358971	c.7471C>T	p.Gln2491Ter	Pathogenic (3*)	2	AA (2)	African American and African (2)
13:32356472:C:T	*BRCA2*	Stop gained	rs80358972	c.7480C>T	p.Arg2494Ter	Pathogenic (3*)	2	EA (1), HA (1)	Non-AJ European (1), Dominican (1)
13:32356550:C:T	*BRCA2*	Stop gained	rs80358981	c.7558C>T	p.Arg2520Ter	Pathogenic (3*)	2	AA (2)	African American and African (1)
13:32357794:CAG:C	*BRCA2*	Frameshift	rs80359672	c.7673_7674delAG	p.Glu2558fs	Pathogenic (3*)	1	HA (1)	Filipino and other Southeast Asian (1)
13:32362626:GCCTTT:G	*BRCA2*	Frameshift	rs80359686	c.7913_7917delTTCCT	p.Phe2638fs	Pathogenic (3*)	1	EA (1)	AJ (1)
13:32363498:AC:A	*BRCA2*	Frameshift	rs80359705	c.8297delC	p.Thr2766fs	Pathogenic (3*)	1	EA (1)	Non-AJ European (1)
13:32370483:TTA:T	*BRCA2*	Frameshift	rs397507984	c.8415_8416delAT	p.Leu2805fs	Pathogenic (3*)	1	AA (1)	African American and African (1)
13:32379800:G:A	*BRCA2*	Missense	rs80359152	c.9004G>A	p.Glu3002Lys	Pathogenic/Likely pathogenic (2*)	2	HA (1), ESA (1)	Dominican (1), Filipino and other Southeast Asian (1)
13:32379913:G:A	*BRCA2*	Synonymous	rs28897756	c.9117G>A	p.Pro3039=	Pathogenic (2*)	1	EA (1)	Non-AJ European (1)
13:32380085:C:T	*BRCA2*	Stop gained	rs80359180	c.9196C>T	p.Gln3066Ter	Pathogenic (3*)	2	AA (2)	African American and African (2)
13:32380135:G:GA	*BRCA2*	Frameshift	rs80359752	c.9253dupA	p.Thr3085fs	Pathogenic (3*)	3	EA (2), AA (1)	AJ (1), Non-AJ European (2)
13:32394726:C:G	*BRCA2*	Stop gained	rs80359200	c.9294C>G	p.Tyr3098Ter	Pathogenic (3*)	1	AA (1)	African American and African (1)
13:32394814:C:T	*BRCA2*	Stop gained	rs80359212	c.9382C>T	p.Arg3128Ter	Pathogenic (3*)	1	M (1)	Non-AJ European (1)
13:32394842:C:CT	*BRCA2*	Frameshift	rs876659435	c.9413dupT	p.Leu3138fs	Pathogenic (3*)	1	EA (1)	Non-AJ European (1)
13:32394863:CTG:C	*BRCA2*	Frameshift	rs80359763	c.9435_9436delGT	p.Ser3147fs	Pathogenic (3*)	1	EA (1)	Non-AJ European (1)

Abbreviations: Hets, heterozygous carriers; AA, African American/African; AJ, Ashkenazi Jewish; EA, European; ESA, East/Southeast Asian; HA, Hispanic/Latino; M, multiple selected; NA, not available; O, other ancestry. cDNA and protein position provided for *BRCA1* ENST00000357654 (NM_007294.3) and *BRCA2* ENST00000380152 (NM_000059.3); Human reference genome build 38 (GRCh38).
