# Supplementary material for: Exome sequencing reveals a high prevalence of BRCA1 and BRCA2 founder variants in a diverse population-based biobank
Source: Genome Med. 2019 Dec 31;12:2. doi: 10.1186/s13073-019-0691-1 (PMC6938627; doi:10.1186/s13073-019-0691-1)
Supplement: Supplementary file 2 — Additional file 2. Banner Author Lists and Contribution Statements. The Charles Bronfman Institute of Personalized Medicine (CBIPM) Genomics Team Banner Author List and Contribution Statements. Regeneron Genetics Center Banner Author List and Contribution Statements. [file 13073_2019_691_MOESM2_ESM.pdf]

ADDITIONAL FILE 2

Exome Sequencing Reveals a High Prevalence of *BRCA1* and *BRCA2* Founder Variants in a Diverse Population-Based Biobank

Noura S. Abul-Husn[1,2,3,4*], Emily R. Soper[1,2†], Jacqueline A. Odgis[1,2†], Sinead Cullina[1,2], Dean Bobo[1,2], Arden Moscati[1,2], Jessica E. Rodriguez[1,2], CBIPM Genomics team[1,2], Regeneron Genetics Center[5], Ruth J.F. Loos[2], Judy H. Cho[2,3,4], Gillian M. Belbin[1,2,3], Sabrina A. Suckiel[1,2], Eimear E. Kenny[1,2,3,4]

[1]The Center for Genomic Health, Icahn School of Medicine at Mount Sinai, New York, NY
[2]The Charles Bronfman Institute for Personalized Medicine, Icahn School of Medicine at Mount Sinai, New York, NY
[3]Department of Medicine, Icahn School of Medicine at Mount Sinai, New York, NY
[4]Department of Genetics and Genomic Sciences, Icahn School of Medicine at Mount Sinai, New York, NY
[5]Regeneron Genetics Center, Tarrytown, New York, NY

[†] These authors contributed equally to this work

***Correspondence:** noura.abul-husn@mssm.edu

The Charles Bronfman Institute of Personalized Medicine (CBIPM) Genomics Team Banner Author List and Contribution Statements

All authors/contributors are listed in alphabetical order.

CBIPM Leadership Team
Noura Abul-Husn, M.D., Ph.D., Erwin Bottinger, M.D., Judy Cho, M.D., Ron Do, Ph.D., Steve Ellis, Omri Gottesman, M.D., Yuval Itan, Ph.D., Eimear Kenny, Ph.D., Ruth Loos, Ph.D., Amanda Merkelson, M.P.H., Girish Nadkarni, M.D., Aniwaa Owusu-Obeng, Pharm.D.

Contribution: All authors contributed to securing funding, study design and oversight.

Sequencing and Lab Operations
Bernadette Liggayu, Amanda Merkelson, M.P.H., Janice Morinigo, Patrick Shanley, Quingbin Song, M.D.

Contribution: All authors are responsible for DNA extraction, sample handling and tracking, and the library information management system.

Clinical Informatics
Noura Abul-Husn, M.D., Ph.D., Lili Chan, M.D., Steve Ellis, Omri Gottesman, M.D., Arden Moscati, Ph.D., Girish Nadkarni, M.D., Rajiv Nadukuru, M.S., Aniwaa Owusu-Obeng, Pharm.D., Tielman Van Vleck, Ph.D.

Contribution: All authors are responsible for analysis needed to produce electronic health record extracted data.

Genome Informatics
Gillian Belbin, Ph.D., Dean Bobo, M.S., Kumardeep Chaudhary, Ph.D., Nathalie Chami, Ph.D., Sinead Cullina, M.S., Ron Do, Ph.D., Aine Duffy, M.S., Amanda Dobbyn, Ph.D., Yuval Itan, Ph.D., Eimear Kenny, Ph.D., Margaret Linan, Carla Marquez-Luna, Ph.D., Arden Moscati, Ph.D., Ha My Vy Thi, Ph.D., Michael Preuss, Ph.D., Cigdem Sevim Bayrak, Ph.D., Stephane Wenric, Ph.D., Ryan Walker, Ph.D., Zhe Wang, Ph.D., Yiming Wu, Ph.D., Lisheng Zhou, Ph.D.

Contribution: All authors are responsible for analysis needed to produce exome and genotype data.

Regeneron Genetics Center Banner Author List and Contribution Statements

All authors/contributors are listed in alphabetical order.

RGC Management and Leadership Team
Goncalo Abecasis, Ph.D., Aris Baras, M.D., Michael Cantor, M.D., Giovanni Coppola, M.D., Aris Economides, Ph.D., John D. Overton, Ph.D., Jeffrey G. Reid, Ph.D., Alan Shuldiner, M.D.

Contribution: All authors contributed to securing funding, study design and oversight. All authors reviewed the final version of the manuscript.

Sequencing and Lab Operations
Christina Beechert, Caitlin Forsythe, M.S., Erin D. Fuller, Zhenhua Gu, M.S., Michael Lattari, Alexander Lopez, M.S., John D. Overton, Ph.D., Thomas D. Schleicher, M.S., Maria Sotiropoulos Padilla, M.S., Karina Toledo, Louis Widom, Sarah E. Wolf, M.S., Manasi Pradhan, M.S., Kia Manoochehri, Ricardo H. Ulloa.

Contribution: C.B., C.F., K.T., A.L., and J.D.O. performed and are responsible for sample genotyping. C.B, C.F., E.D.F., M.L., M.S.P., K.T., L.W., S.E.W., A.L., and J.D.O. performed and are responsible for exome sequencing. T.D.S., Z.G., A.L., and J.D.O. conceived and are responsible for laboratory automation. M.P., K.M., R.U., and J.D.O are responsible for sample tracking and the library information management system.

Genome Informatics
Xiaodong Bai, Ph.D., Suganthi Balasubramanian, Ph.D., Leland Barnard, Ph.D., Andrew Blumenfeld, Gisu Eom, Lukas Habegger, Ph.D., Young Hahn, Alicia Hawes, B.S., Shareef Khalid, Jeffrey G. Reid, Ph.D., Evan K. Maxwell, Ph.D., William Salerno, Jeffrey C. Staples, Ph.D., Ashish Yadav, M.S.

Contribution: X.B., A.H., W.S. and J.G.R. performed and are responsible for analysis needed to produce exome and genotype data. G.E., Y.H., and J.G.R. provided compute infrastructure development and operational support. S.K., S.B., and J.G.R. provide variant and gene annotations and their functional interpretation of variants. E.M., L.B., J.S., A.B., A.Y., L.H., J.G.R. conceived and are responsible for creating, developing, and deploying analysis platforms and computational methods for analyzing genomic data.

Planning, Strategy, and Operations
Marcus B. Jones, Ph.D., Lyndon J. Mitnaul, Ph.D.

Contribution: All authors contributed to the management and coordination of all research activities, planning and execution. All authors contributed to the review process for the final version of the manuscript.
